# MiR-200c-3p Modulates Cisplatin Resistance in Biliary Tract Cancer by ZEB1-Independent Mechanisms

**DOI:** 10.3390/cancers13163996

**Published:** 2021-08-08

**Authors:** Florian Posch, Felix Prinz, Amar Balihodzic, Christian Mayr, Tobias Kiesslich, Christiane Klec, Katharina Jonas, Dominik A. Barth, Jakob M. Riedl, Armin Gerger, Martin Pichler

**Affiliations:** 1 Department of Internal Medicine, Division of Oncology, Medical University of Graz, 8036 Graz, Austria; florian.posch@medunigraz.at (F.P.); felix.prinz@medunigraz.at (F.P.);amar.balihodzic@protonmail.com (A.B.); christiane.klec@medunigraz.at (C.K.); katharina.jonas@medunigraz.at (K.J.); dominik.barth@medunigraz.at (D.A.B.); jakob.riedl@medunigraz.at (J.M.R.); armin.gerger@medunigraz.at (A.G.); 2 Research Unit “Non-Coding RNAs and Genome Editing in Cancer”, Division of Oncology, Medical University of Graz, 8036 Graz, Austria; 3 Institute of Physiology and Pathophysiology, Paracelsus Medical University, 5020 Salzburg, Austria; christian.mayr@pmu.ac.at (C.M.); tobias.kiesslich@pmu.ac.at (T.K.); 4 Department of Internal Medicine I, University Clinics Salzburg, Paracelsus Medical University, 5020 Salzburg, Austria; 5 Department of Experimental Therapeutics, The University of Texas MD Anderson Cancer Center, Houston, TX 77030, USA

**Keywords:** biliary tract cancer, chemoresistance, cisplatin resistance, epithelial–mesenchymal transition, miR-200c-3p, ZEB1

## Abstract

**Simple Summary:**

Biliary tract cancer is a rare malignancy with poor overall survival. The majority of patients are faced with advanced disease stage. Cisplatin-based treatment schedules represent the mainstay of first-line therapeutic strategy, yet only a small portion of patients develop a treatment response. One of the main reasons is acquired drug resistance. Previous studies correlated certain microRNAs (miRNAs), including miR-200c-3p, with drug resistance in various cancer types. However, limited knowledge exists about miR-200c-3p expression and cisplatin resistance in biliary tract cancer. Thus, the main aim of this study was to investigate the influence of miR-200c-3p on the cisplatin resistance in this cancer entity. We demonstrated that miR-200c-3p contributes to cisplatin resistance independently of its known influence on ZEB1 expression.

**Abstract:**

Biliary tract cancer is a major global health issue in cancer-related mortality. Therapeutic options are limited, and cisplatin-based treatment schedules represent the mainstay of first-line therapeutic strategies. Although the gain of survival by the addition of cisplatin to gemcitabine is moderate, acquired cisplatin resistance frequently leads to treatment failures with mechanisms that are still poorly understood. Epithelial–mesenchymal transition (EMT) is a dynamic process that changes the shape, function, and gene expression pattern of biliary tract cancer cells. In this study, we explored the influence of the EMT-regulating miR-200c-3p on cisplatin sensitivity in biliary tract cancer cells. Using gain of function experiments, we demonstrated that miR-200c-3p regulates epithelial cell markers through the downregulation of the transcription factor ZEB1. MiR-200c-3p upregulation led to a decreased sensitivity against cisplatin, as observed in transient overexpression models as well as in cell lines stably overexpressing miR-200c-3p. The underlying mechanism seems to be independent of miR-200c-3p’s influence on *ZEB1* expression, as ZEB1 knockdown resulted in the opposite effect on cisplatin resistance, which was abolished when ZEB1 knockdown and miR-200c-3p overexpression occurred in parallel. Using a gene panel of 40 genes that were previously associated with cisplatin resistance, two (Dual Specificity Phosphatase 16 (*DUSP16*) and Stratifin (*SFN*)) were identified as significantly (>2 fold, *p*-value < 0.05) up-regulated in miR-200c-3p overexpressing cells. In conclusion, miR-200c-3p might be an important contributor to cisplatin resistance in biliary tract cancer, independently of its interaction with ZEB1.

## 1. Introduction

Biliary tract cancer (BTC) is a rare malignancy arising from the epithelium of intrahepatic or extrahepatic bile ducts or the gallbladder [1]. Despite the low incidence in most developed countries, BTC is turning into a major global health issue as the number of new intrahepatic cases is constantly rising [2]. BTC is an aggressive cancer type with poor overall survival. Surgery is the only potential curative treatment, but most patients are diagnosed with locally advanced or metastatic disease [3,4]. Therapeutic options in these situations are limited. Gemcitabine plus cisplatin treatment schedules represent the mainstay of first-line therapy. While this combination, chemotherapy shows superiority and improved survival over gemcitabine alone, and acquired drug resistance leads to a modest 3-month increase in median survival time [5,6,7].

Mechanisms underlying cisplatin resistance in cancer are poorly understood. Previous studies reported the involvement of DNA repair mechanisms, altered cellular accumulation, and drug metabolism [8,9]. In addition, deregulated microRNA (miRNA) expression has been associated with cisplatin resistance in various cancers, including BTC [10,11,12]. miRNAs are a class of small, single-stranded, non-coding, regulatory RNAs with a length of 17–25 nucleotides. Their main role is the post-transcriptional regulation of target mRNAs by binding to their 3′-untranslated region (3′-UTR), leading to their degradation [13,14]. miR-200c-3p is a member of the miR-200 family, which has been reported to be involved in crucial processes such as cell proliferation, invasion, metastasis, apoptosis, and drug resistance [15]. In addition to resistance to paclitaxel and doxorubicin in breast cancer, epidermal growth factor receptor (EGFR) tyrosine kinase inhibitors and radiotherapy in non-small cell lung cancer (NSCLC), and etoposide and topotecan in small cell lung cancer (SCLC) [16,17,18,19,20], alterations in miR-200c-3p expression levels have been reported to be responsible for the resistance to cisplatin in gastric cancer, esophageal cancer, and ovarian cancer [21,22,23].

Multiple studies proved that common molecular mechanisms of miR-200c-3p-related drug resistance are mediated via upregulation of zinc finger E-box binding homeobox (ZEB) family genes [24,25,26,27]. ZEB proteins downregulate E-cadherin, a transmembrane glycoprotein important in the maintenance of cell–cell interactions [28]. Thus, the loss of E-cadherin leads to weakened cell–cell interactions and eventual induction of epithelial–mesenchymal transition (EMT) in cancers [29,30]. EMT has been demonstrated to contribute to cancer cell plasticity by switching between continuous states of cell fate [31], and miRNAs regulating this process might be contributors to cisplatin resistance.

Limited knowledge exists about miR-200c-3p expression and cisplatin resistance in BTC. Therefore, the present study investigated the role of miR-200c-3p in the cisplatin resistance of BTC cells.

## 2. Materials and Methods

### 2.1. Cell Culture

A panel of ten BTC cell lines was used in this study: EGI-1 (ACC 385 [32]) and TFK-1 (ACC 344 [33]) cells were purchased from the German Collection of Microorganisms and Cell Cultures (DSMZ; https://www.dsmz.de, last accessed on 1 August 2021); HuCCT1 (JCRB0425 [34]), HuH28 (JCRB0426 [35]), KKU-055 (JCRB1551), KKU-100 (JCRB1568 [36]), KKU-213 (JCRB1557 [37]), NOZ (JCRB1033 [38]), OCUG-1 (JCRB0191 [39]) and OZ (JCRB1032 [40]) were purchased from the Japanese Collection of Research Bioresources Cell Bank (JCRB; https://cellbank.nibiohn.go.jp, last accessed on 1 August 2021). The immortalized human cholangiocyte cell line MMNK-1 (JCRB1554 [41]) was purchased from JCRB. All cell lines were cultured in L-glutamine-free Dulbecco’s Modified Eagle Medium (DMEM) containing 4.5 g/L glucose (Gibco, Thermo Fisher Scientific, Waltham, MA, USA). Medium was supplemented with 10% fetal bovine serum (FBS; Serana Europe GmbH, Pessin, Germany) and 1% penicillin/streptomycin (Gibco, Thermo Fisher Scientific, Waltham, MA, USA). Cells were cultured at standard conditions (37 °C, 21% O_2_, 5% CO_2_, and 98% humidity).

### 2.2. Transient Transfection

For transient miR-200c-3p overexpression, MMNK-1, OCUG-1, and HuCCT1 cells were seeded in 6-well plates or 96-well plates and transfected with 10 nM mirVana miR-200c-3p mimic (Cat. #: 4464066, Thermo Fisher Scientific, Waltham, MA, USA) or 10 nM mirVana miRNA mimic Negative Control #1 (Cat. #: 4464058, Thermo Fisher Scientific, Waltham, MA, USA). For transient ZEB1 knockdown, cells were seeded in 6-well plates or 96-well plates and transfected with 50 nM siRNA directed against ZEB1 (Hs_ZEB1_2, Cat. #: SI04272492, QIAGEN, Hilden, Germany) or 50 nM AllStars Negative Control siRNA (Cat. #: 1027280, QIAGEN, Hilden, Germany). The transfection procedure was carried out according to the fast-forward protocol of the HiPerFect Transfection Reagent (Cat. #: 301707, QIAGEN, Hilden, Germany). For co-transfection experiments, MMNK-1 cells were seeded into 96-well plates at a density of 1500 cells per well, and transfected with 10 nM mirVana miR-200c-3p mimic (Cat. #: 4464066, Thermo Fisher Scientific, Waltham, Massachusetts, USA) and 50 nM siRNA directed against ZEB1 (Hs_ZEB1_2, Cat. #: SI04272492, QIAGEN, Hilden, Germany), or 10 nM mirVana miRNA mimic Negative Control #1 (Cat. #: 4464058, Thermo Fisher Scientific, Waltham, MA, USA) and 50 nM AllStars Negative Control siRNA (Cat. #: 1027280, QIAGEN, Hilden, Germany) according to the fast-forward protocol of the HiPerFect Transfection Reagent (Cat. #: 301707, QIAGEN, Hilden, Germany). 

### 2.3. Generation of Stable miRNA Overexpression Cell Lines

OCUG-1 cells were seeded into 12-well plates at a density of 100,000 cells per well. After a twenty-four-hour incubation under standard conditions, growth medium was replaced with medium containing 10 μg/mL Polybrene (Cat #: sc-134220, Santa Cruz Biotechnology, Inc., Dallas, TX, USA), 0.5% ViralPlus Transduction Enhancer (Applied Biological Materials Inc., Vancouver, BC, Canada), and either LentimiRa-GFP-hsa-miR-200c-3p Virus (mh15263, Applied Biological Materials Inc., Vancouver, British Columbia, Canada) or Lenti-III-mir-GFP Control Virus (m002, Applied Biological Materials Inc., Vancouver, BC, Canada). Transduced cells were selected with growth medium containing a sublethal concentration of puromycin (Gibco, Thermo Fisher Scientific, Waltham, MA, USA) for up to eight weeks. To further increase miR-200c-3p overexpression levels, transduced cells were sorted according to their GFP expression via flow cytometry using the FACSARia IIIu (BD Biosciences, Franklin Lakes, NJ, USA). Cells with the highest GFP intensity (top 20% GFP signal) were cultured and used for subsequent experiments.

### 2.4. Reverse Transcription Quantitative PCR (RT-qPCR)

Total RNA was isolated from 70 to 90% confluent cells using QIAzol Lysis Reagent (Cat. #: 79306, QIAGEN, Hilden, Germany) according to the manufacturer’s recommendations. Subsequently, 1 μg of total RNA was reversely transcribed with the miScript II RT Kit (Cat. #: 218161, QIAGEN, Hilden, Germany) using the miScript HiFlex buffer to enable quantification of both miRNA and mRNA. qPCR was performed on the LightCycler 480 (Roche, Basel, Switzerland) using the QuantiTect SYBR Green PCR Kit (Cat. #: 204145, QIAGEN, Hilden, Germany). For the quantification of miR-200c-3p, the Hs_miR-200c_1 miScript Primer Assay (Cat. #: MS00003752, QIAGEN, Hilden, Germany) and Hs_RNU6-2_11 miScript Primer Assay (Cat. #: MS00033740, QIAGEN, Hilden, Germany) were used, with RNU6-2 serving as a reference gene. For mRNA quantification, expression levels of Keratin 8 (*KRT8*), E-cadherin (*CDH1*), N-cadherin (*CDH2*), Collagen Type III Alpha 1 Chain (*COL3A1*), Vimentin (*VIM*), zinc finger E-box binding homeobox 1 (*ZEB1*), zinc finger E-box binding homeobox 2 (*ZEB2*), and genes that have previously been associated with cisplatin resistance (see Table S1) were normalized to the mean expression of *GAPDH* and *U6*. Primer sequences are listed in Table S2. Differences in gene expression were evaluated by the ΔΔCt method.

### 2.5. Immunoblot Analysis

Cells were lysed in radioimmunoprecipitation assay (RIPA) buffer supplemented with 2% Protease Inhibitor Cocktail (Thermo Fisher Scientific, Waltham, Massachusetts, USA). Thirty micrograms of total protein was mixed with 4x Laemmli buffer containing 10% β-mercaptoethanol and denaturized at 95 °C for 10 min. Proteins were separated on 4–15% Mini-PROTEAN^®^ TGX Stain-free™ Gels (Bio-Rad Laboratories, Inc., Hercules, CA, USA) and transferred to a 0.45 µm nitrocellulose membrane (Bio-Rad Laboratories, Inc., Hercules, CA, USA). Membranes were blocked with 5% milk powder in Tris-buffered saline (TBS) containing 0.1% Tween^®^ 20 (5% M-TBS-T) for 1–5 h before incubation with primary antibody targeted at ZEB1 (TCF8/ZEB1 (D80D3) Rabbit mAb, #702743, Cell Signaling Technology^®^, Danvers, Massachusetts, USA; diluted 1:1000 in 2.5% M-TBS-T) at 4 °C for 16 h. Subsequently, membranes were incubated with the corresponding secondary horseradish peroxidase (HRP)-conjugated antibody (Peroxidase AffiniPure Goat Anti-Rabbit IgG antibody, 111-035-144, Jackson ImmunoResearch Laboratories, Inc., West Grove, PA, USA; diluted 1:10,000 in 2.5% M-TBS-T) for 1 h at room temperature (RT). Membranes were incubated with SuperSignal^®^ West Femto PLUS Chemiluminescent Substrate (Bio-Rad Laboratories, Inc., Hercules, CA, USA) and imaged on a ChemiDoc™ Touch (Bio-Rad Laboratories, Inc., Hercules, CA, USA). To verify equal protein loading, membranes were stripped and re-probed for Cofilin (Anti-Cofilin antibody, ab42824, Abcam, Cambridge, UK; diluted 1:5000 in 2.5% M-TBS-T). Uncropped Western blot images can be found at Figure S5.

### 2.6. Cisplatin Sensitivity Assay

MMNK-1, OCUG-1, and HuCCT1 cells were seeded into 96-well plates at densities of 1500, 500, and 3500 cells per well, respectively, and transfected according to the HiPerFect fast-forward protocol (QIAGEN, Hilden, Germany). Twenty-four hours later, standard growth medium was exchanged with fresh growth medium containing cisplatin in various concentrations (0–50 µM). Forty-eight hours after the addition of cisplatin, 100 µL of medium was removed and 10 µL Cell Proliferation Reagent WST-1 (Cat. #: 11644807001, Roche, Basel, Switzerland) was added to each well. After a 180 min incubation at standard conditions, absorbances at 450 nm and 620 nm (reference wavelength) were measured on the SPECTROstar Omega (BMG LABTECH GmbH, Ortenberg, Germany). Results were used for the IC50 calculations via ordinary least-squares regression with outlier removal following the ROUT method [42].

### 2.7. Statistical Analysis

All experiments were performed at least three independent times. Data are presented as mean ± standard deviation (SD). Statistical analyses were performed using the GraphPad Prism 5.0 software (GraphPad Software, San Diego, CA, USA). The unpaired two-tailed t-test and one-way ANOVA with Tukey’s Post Hoc test were used when applicable. A *p* value of less than 0.05 was regarded as statistically significant. * *p* < 0.05, ** *p* < 0.01, *** *p* < 0.001.

## 3. Results

### 3.1. MiR-200c-3p and Its Influence on EMT Markers in Immortalized Cholangiocytes, Biliary Tract Cancer Cell Lines, and Human Tissue

**Figure 1:**
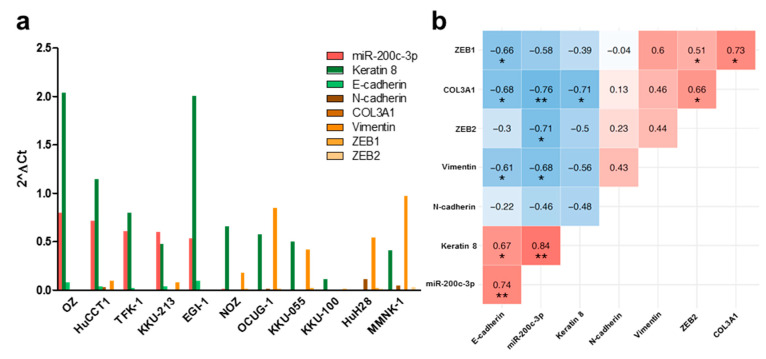
Endogenous expression levels of miR-200c-3p correlates with the expression of EMT markers in immortalized cholangiocytes and biliary tract cancer cell lines. (**a**) Expression levels of miR-200c-3p and various EMT markers were analyzed in immortalized cholangiocytes (MMNK-1 cells) and 10 BTC cell lines via RT-qPCR. (**b**) Spearman rank correlation matrix of endogenously expressed miR-200c-3p and EMT markers in MMNK-1 cells and BTC cell lines. Positive correlations are depicted in shades of red, negative correlations in shades of blue. Correlation coefficient and statistical significance indicators are shown in the respective tiles. * *p* < 0.05, ** *p* < 0.01.

**Figure 2:**
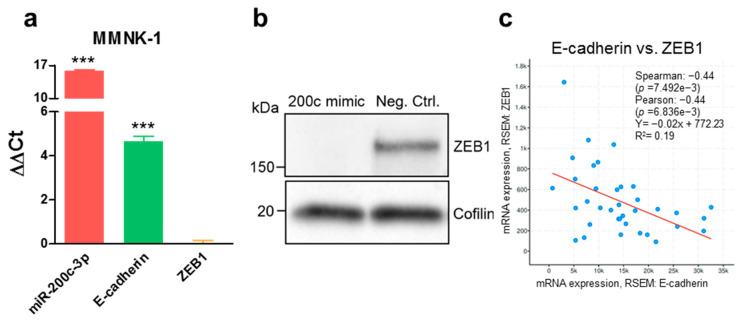
Transient miR-200c-3p overexpression induces an epithelial phenotype through the interaction with E-cadherin and ZEB1. (**a**) MMNK-1 cells were transiently transfected with 10 nM mirVana miR-200c-3p mimic, and the expression levels of miR-200c-3p, E-cadherin (*CDH1*), and *ZEB1* were compared to those of respective cells transfected with 10 nM mirVana mimic negative control. Positive values represent an overexpression. A value of 0 indicates that there was no difference between cells transfected with miR-200c-3p mimic and mimic negative control. Unpaired two-tailed t-test was used to evaluate statistical significance. *** *p* < 0.001. (**b**) ZEB1 protein levels were compared between miR-200c-3p mimic-transfected MMNK-1 cells and Neg. Ctrl.-transfected MMNK-1 cells. Cofilin was used as loading control. (**c**) In silico correlation analysis of E-cadherin (*CDH1*) and *ZEB1* expression levels in 36 human BTC tissue samples from The Cancer Genome Atlas.

In an attempt to better understand the relationship between miR-200c-3p and the epithelial–mesenchymal transition status in BTC, we analyzed the endogenous expression levels of miR-200c-3p and several epithelial (Keratin 8 (*KRT8*), E-cadherin (*CDH1*)) and mesenchymal (N-cadherin (*CDH2*), Collagen Type III Alpha 1 Chain (*COL3A1*), Vimentin (*VIM*), *ZEB1*, *ZEB2*) gene expression markers in immortalized cholangiocytes and BTC cell lines. Based on these endogenous expression levels, cell lines with a predominantly epithelial expression profile (high miR-200c-3p, high *CDH1*, and low *ZEB1*) or a predominantly mesenchymal expression profile (low miR-200c-3p, low *CDH1*, and high *ZEB1*) were determined (Figure 1a). A significant correlation between miR-200c-3p and epithelial gene expression markers was confirmed (Figure 1b). Additionally, we functionally confirmed the relationship between *ZEB1*, *CDH1*, and miR-200c-3p expression in MMNK-1 cells by gain of function experiments. After ectopic overexpression of miR-200c-3p, an increased expression of *CDH1* on RNA level (Figure 2a) and a decreased expression of ZEB1 on protein level (Figure 2b) were observed. The negative correlation between *ZEB1* and *CDH1* was further validated in 36 human BTC tissue samples of The Cancer Genome Atlas datasets (Figure 2c).

### 3.2. Ectopic MiR-200c-3p Overexpression Increases Cisplatin Resistance in Immortalized Cholangiocytes and Biliary Tract Cancer Cells

**Figure 3:**
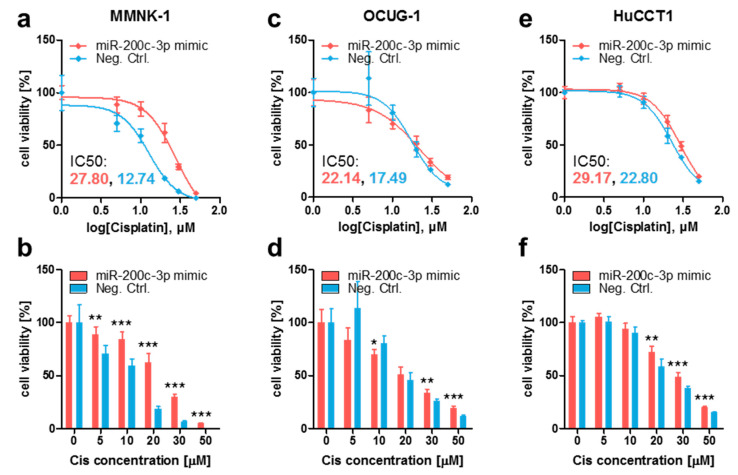
Transient miR-200c-3p overexpression renders immortalized cholangiocytes and BTC cells more resistant to cisplatin. (**a**,**b**) MMNK-1, (**c**,**d**) OCUG-1, and (**e**,**f**) HuCCT1 cells were transfected with 10 nM mirVana miR-200c-3p mimic (miR-200c-3p mimic) or mirVana mimic Negative Control (Neg. Ctrl.) for 24 h and subsequently treated with various concentrations of cisplatin (Cis). After 48 h, cell viability was assessed by adding WST-1 reagent and measuring absorbances at 450 nm and 620 nm (reference wavelength). Cells treated with cisplatin were normalized to untreated (0 μM) cells (set to 100%). (**a**,**c**,**e**) Ordinary least-squares regression with outlier removal following the ROUT method was used to determine the inhibitory concentration 50% (IC50) values. (**b**,**d**,**f**) Unpaired two-tailed t-test was used to evaluate statistical significance. * *p* < 0.05, ** *p* < 0.01, *** *p* < 0.001.

Cisplatin is a mainstay of therapeutic strategies and has significantly improved the survival of metastatic BTC patients in recent years [5]. Since there seems to be a link between the EMT status of cancer cells and their sensitivity to cisplatin, and because miR-200c-3p expression shows a tendency to influence overall survival in patients suffering from cholangiocarcinoma (Figure S1), we sought to determine if the EMT-regulating miR-200c-3p affects the cisplatin sensitivity of BTC cells. Therefore, we ectopically overexpressed miR-200c-3p by transfecting MMNK-1, OCUG-1, and HuCCT1 cells with miR-200c-3p mimic and mimic Negative Control (Neg. Ctrl.). After confirming a dose-dependent and time-dependent effect of cisplatin (Figure S2), cisplatin sensitivity was compared between miR-200c-3p mimic-transfected and Neg. Ctrl.-transfected cells. In all three cell lines, miR-200c-3p overexpression rendered cells more resistant to cisplatin when compared to their control counterparts, which was made apparent by the increased inhibitory concentration 50% (IC50) values (Figure 3a,c,e) and in the direct viability comparison (Figure 3b,d,f).

### 3.3. Stable Endogenous MiR-200c-3p Overexpression Increases Cisplatin Resistance in Biliary Tract Cancer Cells

**Figure 4:**
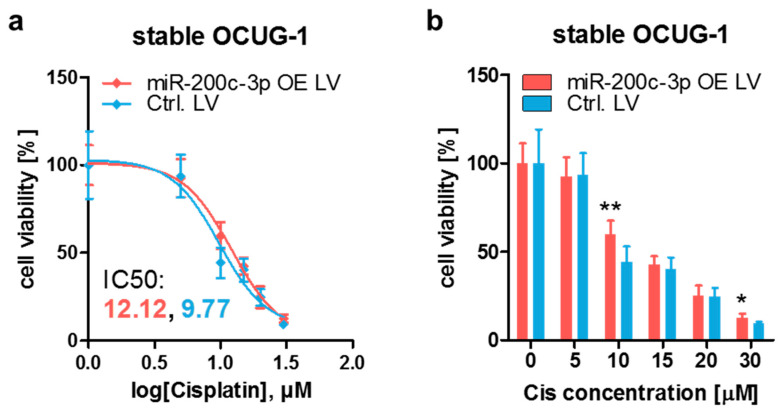
Stable miR-200c-3p overexpression results in an increased cisplatin resistance in OCUG-1 cells. (**a**) IC50 determination and (**b**) direct comparison of stable miR-200c-3p overexpression OCUG-1 cells (miR-200c-3p OE LV) and OCUG-1 control cells (Ctrl. LV). Cells were treated with various concentrations of cisplatin (Cis). After 48 h, cell viability was assessed by adding WST-1 reagent and measuring the absorbance at 450 nm and 620 nm (reference wavelength). Cells treated with cisplatin were normalized to untreated (0 μM) cells (set to 100%). (**a**) Ordinary least-squares regression with outlier removal following the ROUT method was used to determine the IC50 values of the respective cells. (**b**) Unpaired two-tailed t-test was used to evaluate statistical significance. * *p* < 0.05, ** *p* < 0.01.

To corroborate the results of the transient overexpression setting, stable endogenous miR-200c-3p overexpression OCUG-1 cells were generated by lentiviral transduction (Figure S3). In comparison to Control cells (Ctrl. LV), miR-200c-3p overexpression cells (miR-200c-3p OE LV) were less sensitive to cisplatin, confirming the results from the transient overexpression models (Figure 4).

### 3.4. ZEB1 Knockdown Decreases Cisplatin Resistance of MMNK-1 Cells, Rescue Experiments and Cisplatin-Resistance Associated Gene Expression Pattern

**Figure 5:**
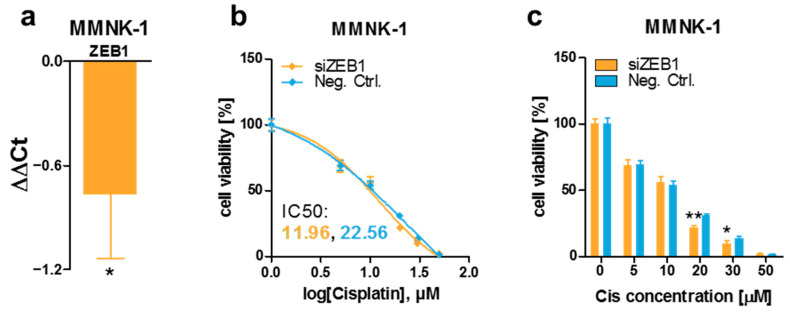
Transient ZEB1 knockdown leads to a decreased cisplatin resistance in MMNK-1 cells. (**a**) MMNK-1 cells were transfected with 50 nM ZEB1 siRNA (siZEB1) or Allstars Neg. Ctrl. (Neg. Ctrl.), and the *ZEB1* expression level was compared via the ΔΔCt method. A negative value represents a downregulation; a value of 0 indicates that there was no difference between siZEB1-transfected cells and Neg. Ctrl.-transfected cells. Unpaired two-tailed t-test was used to evaluate statistical significance. (**b**) IC50 determination via ordinary least-squares regression with outlier removal following the ROUT method and (**c**) direct comparison of transient ZEB1 knockdown and control conditions in MMNK-1 cells. Cells were transfected with 50 nM ZEB1 siRNA (siZEB1) or AllStars Neg. Ctrl. (Neg. Ctrl.) and subsequently treated with various concentrations of cisplatin (Cis). After 48 h, cell viability was assessed by adding WST-1 reagent and measuring absorbance at 450 nm and 620 nm (reference wavelength). Cells treated with cisplatin were normalized to untreated (0 μM) cells (set to 100%). Unpaired two-tailed t-test was used to evaluate statistical significance. * *p* < 0.05, ** *p* < 0.01.

**Figure 6:**
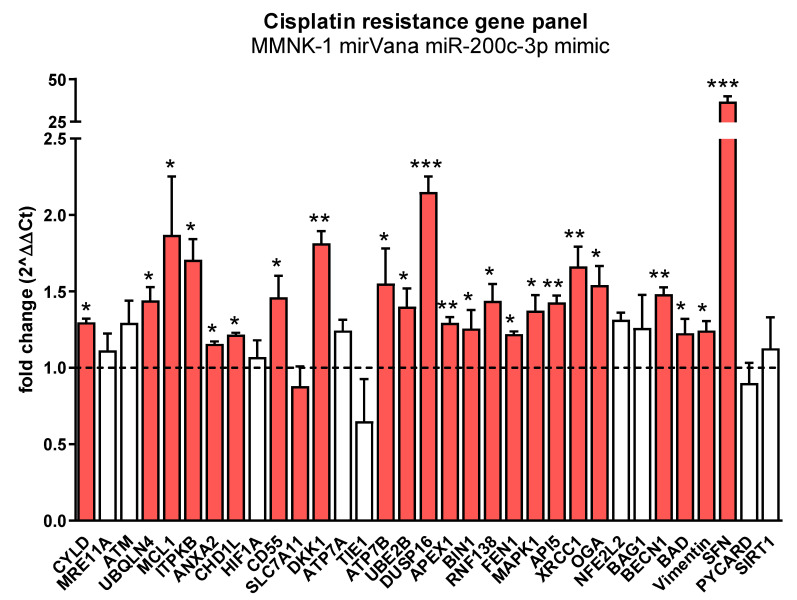
The cisplatin conferring genes *DUSP16* and *SFN* are significantly (>2 fold, *p*-value < 0.05) up-regulated in miR-200c-3p overexpressing cells. MMNK-1 cells were transfected with 10 nM mirVana miR-200c-3p mimic or 10 nM mirVana miRNA mimic Negative Control. After 48 h, total RNA was isolated from 70 to 90% confluent cells using QIAzol Lysis Reagent. Reverse Transcription Quantitative PCR (RT-qPCR) of cisplatin-resistance-conferring genes was performed, and gene expression was normalized to GAPDH and U6. Differences in gene expression were evaluated by the ΔΔCt method. Unpaired two-tailed t-test was used to evaluate statistical significance. * *p* < 0.05, ** *p* < 0.01, *** *p* < 0.001.

In order to explore if miR-200c-3p exerts its effect on cisplatin sensitivity by downregulating ZEB1, or in a ZEB1-independent manner, we decreased ZEB1 expression levels in MMNK-1 cells by siRNA-mediated knockdown (Figure 5a) and evaluated the changes in cisplatin sensitivity. In contrast to miR-200c-3p-mediated effects, ZEB1 knockdown resulted in a decreased cisplatin resistance in MMNK-1 cells, indicating that different miR-200c-3p-regulated mechanisms are responsible for the observed alterations in cisplatin sensitivity (Figure 5b,c). In order to explore whether the effect of ZEB1 knockdown can be reversed by miR-200c-3p overexpression, we performed a co-transfection experiment with simultaneous transfection of siRNA against ZEB1 and miR-200c-3p mimics. As shown in Figure S4, the effect of ZEB1 knockdown was rescued by co-transfection of miR-200c (IC50 10.6 versus 11.6, *p* = 0.38). In order to identify some possible mechanisms of the miR-200c-3p effects, we measured the expression of about 40 genes that were previously associated with cisplatin resistance. As shown in Figure 6, several genes were significantly differentially expressed, including two with greater than two-fold up-regulation (*DUSP16* and *SFN*).

## 4. Discussion

BTC continues to be an aggressive cancer type with poor outcome and exhibits a low response rate to the cisplatin-based first-line treatment standard. Therefore, understanding the underlying mechanism behind cisplatin-based resistance is of utmost importance for the improvement of current treatment schedules. Emerging evidence suggests that alterations in miRNA expression levels significantly contribute to this resistance issue. For instance, miR-1249 drives cisplatin resistance via the expansion of cancer stem cell populations through Wnt pathway deregulation [12]. Downregulated miR-145 and miR-199a-3p diminish the cisplatin-induced expression of multidrug resistance associated protein 1 (MRP1) and multidrug resistant protein 1 (MDR1) in gallbladder cancer [43,44]. miR-31 contributes to cisplatin resistance in gallbladder cancer via the anti-apoptotic Src/Akt/Bax/Bcl-2 signaling pathway [45]. Similarly, reduced expression of miR-125b-5p contributes to cisplatin resistance through upregulated Bcl-2 expression [46].

In our study, we focused on miR-200c-3p, a previously described factor that is centrally involved in EMT. Furthermore, miR-200c-3p is a member of the miR-200 family and has been reported to be abnormally expressed in various cancers. It is significantly downregulated in prostate cancer, nephroblastoma, breast cancer, and gastric cancer [16,47,48,49,50]. On the other hand, miR-200c-3p is upregulated and leads to worse clinicopathological features in several other cancer entities, such as nasopharyngeal carcinoma, oral squamous cell carcinoma (OSCC), colorectal cancer (CRC), ovarian cancer, small cell lung cancer (SCLC), and endometrial cancer [20,51,52,53,54,55]. Involvement of this miRNA in the development of drug resistance in different cancers has been reported [16,17,18,19,20]. A common molecular mechanism of miR-200c-3p-induced general drug resistance is mediated via the induction of EMT. One study has shown an interaction between long-noncoding RNA ATB (*lncATB*) and miR-200c in breast cancer cells. *LncATB* acts as a competing endogenous RNA (ceRNA) sponging miR-200c. The resulting downregulation of miR-200c leads to restored expression of the transcription factor TWIST1, which further results in EMT-mediated trastuzumab resistance [26,56]. Similarly, an interaction between long-noncoding RNA metastasis-associated lung adenocarcinoma transcript 1 (*MALAT-1*) and miR-200c-3p was reported in pancreatic cancer. *MALAT-1* functions as a ceRNA to suppress miR-200c-3p, leading to the upregulation of *ZEB1* expression [27]. *Circular RNA* hsa_circ_001783 sponges miR-200c-3p in breast cancer, which leads to the upregulation of miR-200c-3p target genes *ZEB1*, *ZEB2*, and *ETS1* [24]. *ZEB2* is a direct target of miR-200c-3p that is downregulated in prostate cancer, which results in EMT [25]. Alternatively, miR-200c-3p modulates the expression of metabolic enzyme cytochrome P450 1B1 (CYP1B1) and transcription factor *SOX2* in renal cell carcinoma and breast cancer, respectively. These effects contribute to the resistance to taxanes [16,57].

However, limited knowledge exists about miR-200c-3p expression and cisplatin-resistance in BTC. Two studies documented lower expression in intrahepatic BTC and gallbladder cancer compared to normal tissue [58,59]. Shen et al. have shown that miR-200c-3p could be an efficient biomarker for BTC as serum levels of exosomal miR-200c-3p positively correlate with the rate of BTC progression [60]. Li et al. found that long intergenic non-protein coding RNA 667 (*LINC00667*) functions as a ceRNA for miR-200c-3p in BTC. As a consequence of this miR-200c-3p downregulation, overexpressed *LINC00667* upregulates pyruvate dehydrogenase kinase 1 (PDK1) to promote the development of BTC through the enhanced proliferation, migration, invasion and EMT of BTC cells [61]. Nevertheless, no study has yet investigated the role of miR-200c-3p in cisplatin resistance in BTC. Due to the well-established association between miR-200c-3p and EMT-mediated chemoresistance in various cancers, the connection between the miR-200 family and EMT in BTC [62], as well as the link between EMT and overall cisplatin resistance [63], we investigated this pathway in BTC.

First of all, our study was conducted to confirm the relationship between miR-200c-3p and the EMT status in BTC. EMT is the process during which cancer cells lose epithelial features, and reorganize their cytoskeletal architecture and cell shape to acquire a mesenchymal phenotype. This process leads to enhanced cell motility and eventual dissemination [64]. Based on the endogenous expression levels of miR-200c-3p and several epithelial and mesenchymal genes, we could ascribe either a predominantly epithelial expression profile (high miR-200c-3p, high E-cadherin, and low ZEB1) or a predominantly mesenchymal expression profile (low miR-200c-3p, low E-cadherin, and high ZEB1) to different BTC cell lines and found a correlation between the expression of miR-200c-3p and EMT marker genes. Furthermore, miR-200c-3p overexpression experiments resulted in an increased expression of E-cadherin (*CDH1*) on RNA level and a decreased ZEB1 protein level. Thus, our results are in accordance with the already well-described relationship between miR-200c-3p and EMT-associated genes such as *ZEB1*.

ZEB1 is a transcriptional factor involved in the regulation of embryonic development and cancer progression. It plays an important role in the regulation of DNA damage repair, cancer cell differentiation, and metastasis. ZEB1 directly binds to the *CDH1* promoter, thereby suppressing transcription of the *CDH1* gene, which encodes for E-cadherin. E-cadherin is a transmembrane glycoprotein whose main function is to form structures that establish and maintain cell–cell interactions. With the loss of E-cadherin, cell–cell interactions are attenuated or completely lost, leading to EMT [28]. In addition, increasing evidence suggests that ZEB1-mediated EMT is an important process affecting the cancer sensitivity to chemotherapeutics [65,66]. Interestingly, we found that miR-200c-3p mediates cisplatin resistance in BTC, and this apparently occurs independently of its suppressive effect on ZEB1, as knockdown of ZEB1 could not recapitulate the increased cisplatin sensitivity seen after miR-200c-3p overexpression. Our gene expression panel of previously reported cisplatin-resistance-conferring genes included *DUSP16* and *SFN*; both proteins have previously been reported to contribute to cisplatin resistance when upregulated [67,68]. Thus, though not comprehensively characterized in our study, these genes may contribute to the effects described herein in this manuscript. 

## 5. Conclusions

In conclusion, the present study is the first report that miR-200c-3p contributes to cisplatin resistance in BTC. Moreover, we found that this cisplatin resistance is mediated via ZEB1-independent mechanisms. Further studies are necessary to elucidate detailed molecular mechanisms.

## Data Availability

Not applicable.

## Supplementary Materials

The following are available online at https://www.mdpi.com/article/10.3390/cancers13163996/s1, Figure S1: Overall survival based on miR-200c-3p expression in cholangiocarcinoma patients, Figure S2: Cisplatin inhibits cell proliferation in a time- and dose-dependent manner, Figure S3: Stable miR-200c-3p overexpression OCUG-1, Figure S4: miR-200c-3p reverses the effect of ZEB1 knockdown, Figure S5: Uncropped Figure 2B, Table S1: Cisplatin resistance gene panel with references, Table S2: Primer sequences for RT-qPCR analysis of mRNA expression.
