# Supplementary material for: MiR-200c-3p Modulates Cisplatin Resistance in Biliary Tract Cancer by ZEB1-Independent Mechanisms
Source: Cancers (Basel). 2021 Aug 8;13(16):3996. doi: 10.3390/cancers13163996 (PMC8392278; doi:10.3390/cancers13163996)
Supplement: Supplementary file 1 [file cancers-13-03996-s001.zip › cancers-1318227-supplementary.pdf]

cancers

Supplementary Material: MiR-200c-3p Modulates Cisplatin Resistance in Biliary Tract Cancer by ZEB1-Independent Mechanisms

Florian Posch, Felix Prinz, Amar Balihodzic, Christian Mayr, Tobias Kiesslich, Christiane Klec, Katharina Jonas, Dominik A. Barth, Jakob M. Riedl, Armin Gerger and Martin Pichler

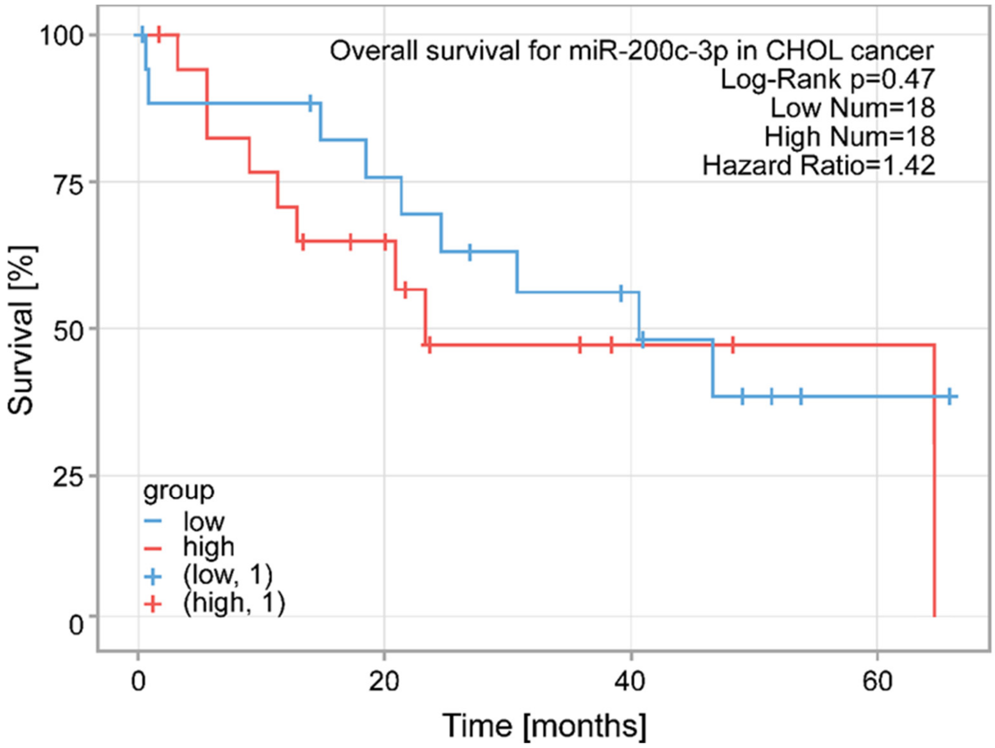

Figure S1: Overall survival based on miR-200c-3p expression in cholangiocarcinoma patients. Patients suffering from cholangiocarcinoma were divided into low miR-200c-3p expression (blue) and high miR-200c-3p expression (red) groups and overall survival in percent was plotted against the time in months. Data was collected from TCGA dataset.

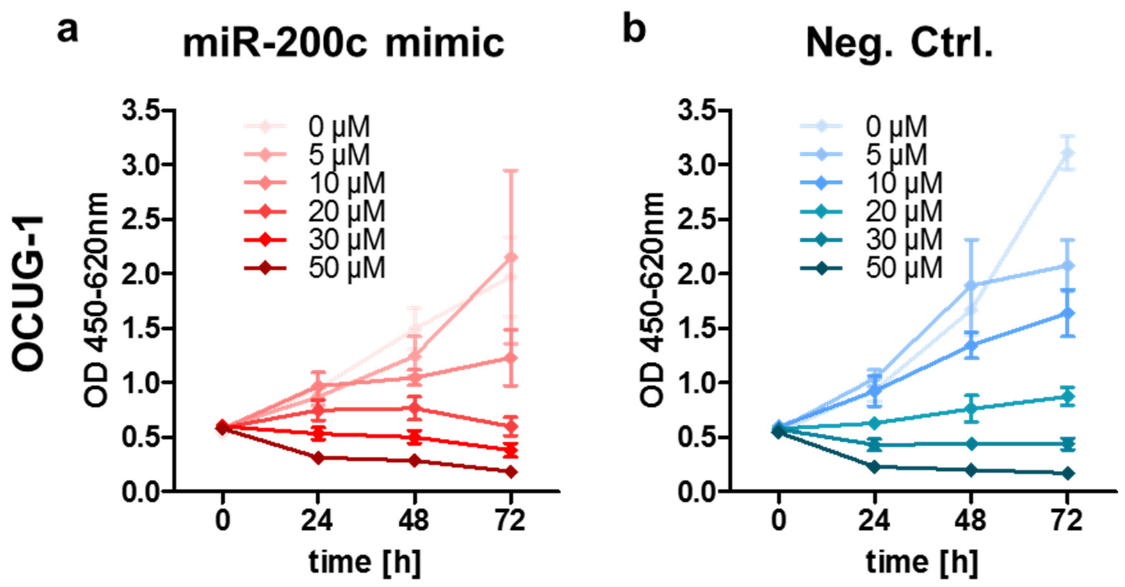

Figure S2: Cisplatin inhibits cell proliferation in a time- and dose-dependent manner. OCUG-1 cells were transfected with (a) 10 nM mirVana miR-200c-3p mimic or (b) mirVana mimic negative control and treated with various concentrations of cisplatin (0-50 µM) over a duration of 96 h. Proliferation was assessed in intervals of 24 h by WST-1 reagent addition and photometric measurement at 450 nm and 620 nm as reference wavelength.

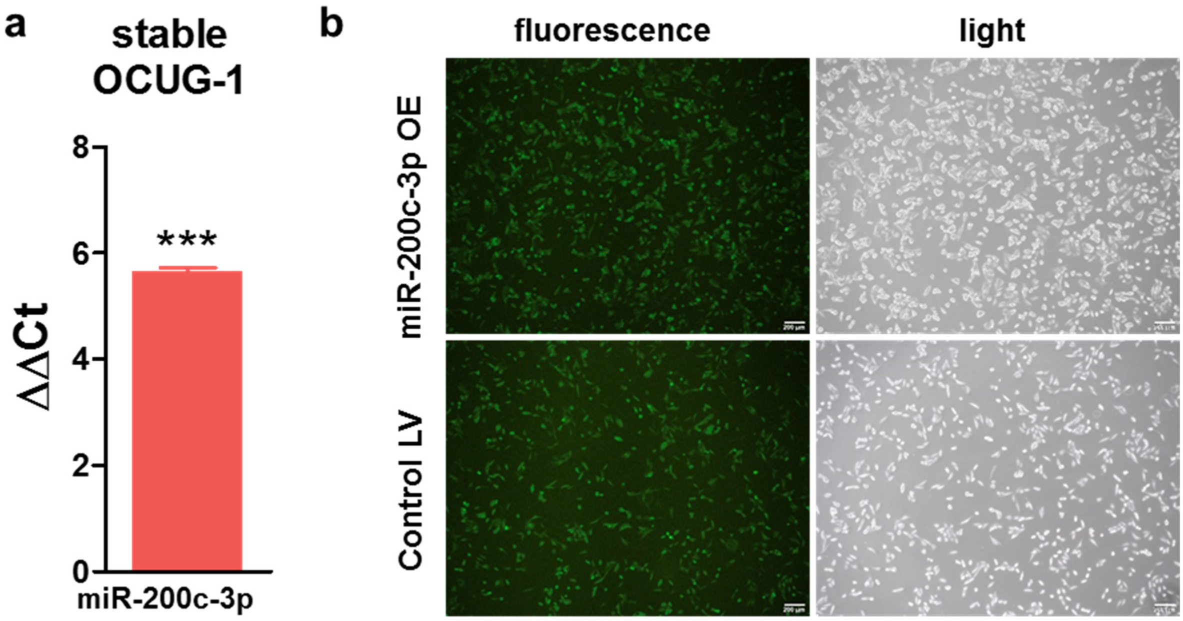

Figure S3: Stable miR-200c-3p overexpression OCUG-1. (a) OCUG-1 cells were transduced with lentiviruses harboring a miR-200c-3p overexpression vector (miR-200c-3p OE) or an empty control vector (Control LV). After selection process, expression level of miR-200c-3p was compared between OCUG-1 miR-200c-3p OE cells and OCUG-1 Control LV cells. Positive values represent and overexpression. A value of 0 means no difference. Unpaired two-tailed t-test was used to evaluate statistical significance. ***P<0.001. (b) Representative microscopic images (fluorescence – GFP, light) of generated OCUG-1 miR-200c-3p OE cells and Control LV cells. 10x magnification was used.

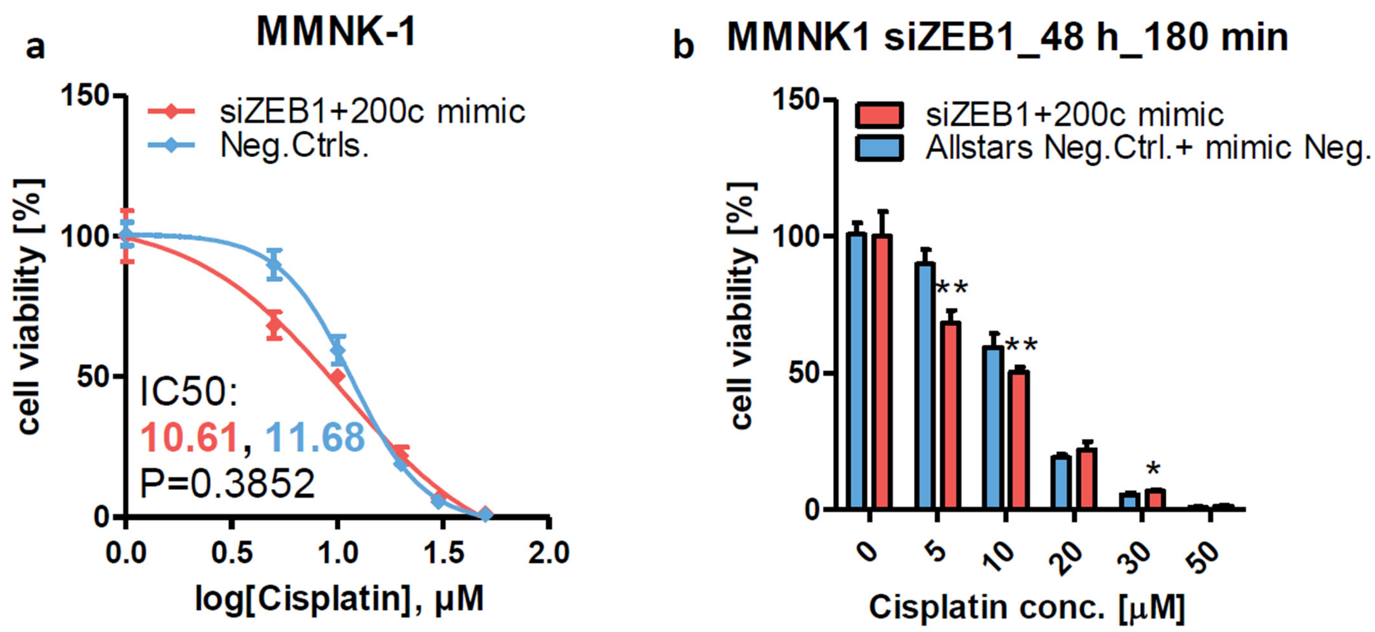

Figure S4: miR-200c-3p reverses the effect of ZEB1 knockdown. (a,b) MMNK-1 cells were co-transfected with 10nM mirVana miR-200c-3p mimic (miR-200c-3p mimic) and 50nM siRNA directed against ZEB1 (siZEB1) or mirVana mimic Negative Control and 50nM siRNA AllStars Negative Control (Neg. Ctrls.). Cells were subsequently treated with various concentrations of cisplatin. After 48 hours, cell viability was assessed by adding WST-1 reagent and measuring the absorbance at 450 nm. 620 nm was measured as reference wavelength. Cells treated with cisplatin were normalized to untreated (0 µM) cells (set to 100%). (a) Ordinary least-squares regression with outlier removal following the ROUT method was used to determine the IC50 values of the respective cells. (b) Unpaired two-tailed t-test was used to evaluate statistical significance.

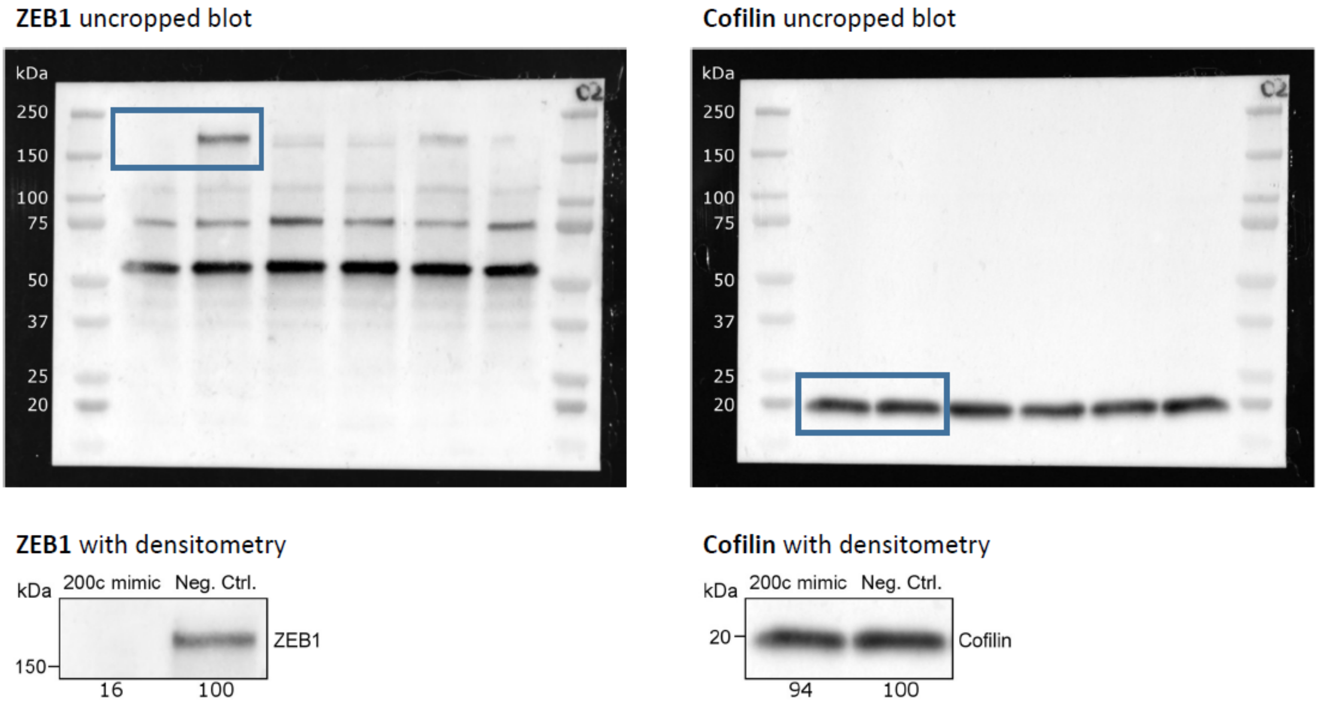

Figure S5: Uncropped Figure 2B.

Table S1: Cisplatin resistance gene panel with references.

GENE	REFERENCE (doi)	GENE	REFERENCE (doi)
**UBQLN4**	10.1002/1878-0261.12929	**PTGER3**	10.1016/j.ebiom.2018.11.045
**MRE11A**		**DKK1**	10.1186/s12885-015-1635-9
**ITPKB**	10.1172/JCI124550	**TIE1**	10.1038/s41598-018-31069-2
**MAST1**	10.1016/j.ccell.2018.06.012; 10.1172/JCI125963	**UBE2B (=RAD6B)**	10.1016/j.bbadis.2019.165561
**CHD1L**	10.1038/s41419-019-1371-1	**APEX1**	10.3892/or.2019.7345
**HIF1A**	10.1016/j.canlet.2016.01.009	**RNF138**	10.1080/15384047.2018.1480293
**CD55**	10.1084/jem.20170438	**MAPK1 (=ERK)**	10.1158/1078-0432.CCR-18-4145
**SLC7A11**	10.18632/oncotarget.12356	**CYLD**	10.3390/ijms20205194
**ATP7A**	10.1016/j.bbadis.2015.12.005;	**CD133**	10.1177/1533033819864311;
**ATP7B**	10.18632/oncotarget.12992		10.1177/1933719113497281
**DUSP16**	10.1038/s41467-021-22638-7	**NFE2L2 (=Nrf2)**	10.1016/j.urolonc.2014.02.006
**BIN1**	10.1074/jbc.RA118.005699	**BAG1**	10.1186/s12967-017-1289-2
**FEN1**	10.1002/1878-0261.12058	**BECN1**	10.2147/DDDT.S126464
**API5**	10.1038/emm.2017.130	**BAD**	10.1042/CBI20110329
**XRCC1**	10.1038/cddis.2014.27	**VIM**	10.18632/oncotarget.9970
**DUOXA1**	10.1016/j.canlet.2018.04.029	**HMGB1**	10.1089/dna.2016.3360
**ATM**	10.1080/15384047.2017.1345391	**RUNX3**	10.1111/1759-7714.12370

**MCL1**		**SFN (=14-3-3σ)**	10.1002/cbin.10006
**ANXA2**	10.1186/s13046-017-0594-1	**PYCARD (=ASC)**	10.1620/tjem.244.133
**AKR1C1**	10.1186/s13046-019-1256-2	**SIRT1**	10.26355/eurrev_201807_15510
**LRRC8A**	10.1152/ajpcell.00256.2015	**H19**	10.1038/srep26093; 10.1002/cam4.1871
**HOXB13**	10.7150/thno.29463		

Table S2: Primer sequences for RT-qPCR analysis of mRNA expression.

PRIMER NAME	SEQUENCE (5′ → 3′)
UBQLN4_fwd	CTACTGCGTCCATACTCTCTGG
UBQLN4_rev	CTCCATGAAGTTGGCAGAGC
MRE11A_fwd	GGAAGAGGTCGAAGAGGTGG
MRE11A_rev	CAGACCAGTGTCTGCTCTTCC
ITPKB_fwd	AACATGGTGCACTGGTCTCC
ITPKB_rev	GCTGCCTTGAAACTCCCTGC
MAST1_fwd	GGAGCTGATCCTTAAGAGTGGC
MAST1_rev	TCCTCCGAGCCATTTTAGCC
CHD1L_fwd	TTACCAGTCCTTCTGCCTGC
CHD1L_rev	GTGGCCAGAGTCATCTACGC
HIF1A_fwd	TCTGCAACATGGAAGGTATTGC
HIF1A_rev	AGCACCAAGCAGGTCATAGG
CD55_fwd	CGTCTTCTATCTGGGCACACG
CD55_rev	TTGGCTAAGTCAGCAAGCCC
SLC7A11_fwd	ACCTTTTGCAAGCTCACAGC
SLC7A11_rev	GTTGAGGTAAAACCAGCCAGC
ATP7A_fwd	GACGTACTGATTGTGCTGGC
ATP7A_rev	CCTCTGATGTTTTGCCCTTTGC
ATP7B_fwd	TCCCCACAATCAACCAGAGC
ATP7B_rev	AACACGGAGAGAACACCAGC
DUSP16_fwd	GCCGTGACAACTTTCGTTTCC
DUSP16_rev	CACTGGACTGAAAGCTTTTGGGG
BIN1_fwd	TTCCCCGACATCAAGTCACG
BIN1_rev	CTCGGCCTTGGCAATTTTGG
FEN1_fwd	AGAAGGGAGAGCGAGCTTAGG
FEN1_rev	ACACAGAGGAGGGATGACTGG
API5_fwd	CACCATGCCGACAGTAGAGG
API5_rev	AGGCATCTTTATGCTGGCCC
XRCC1_fwd	GGGAGCAAGACTATGAGGTCC
XRCC1_rev	CTTGTCAGGCCCAAACATGC
DUOXA1_fwd	CCAAGGCATACTATCGCCCC
DUOXA1_rev	CAATCTGGACTCCTTCCCCG
ATM_fwd	AACGGAGAAAAGAAGCCGTGG
ATM_rev	ACACACATCACTGTCACTGC
MCL1_fwd	GAGGAGGACGAGTTGTACCG
MCL1_rev	TCCACAAACCCATCCTTGG
ANXA2_fwd	ACACATCTGGTGACTTCCGC
ANXA2_rev	GCGTCATAGAGATCCCGAGC
AKR1C1_fwd	TATGCGCCTGCAGAGGTTCC
AKR1C1_rev	GGAAGCCAGCTTCAATTGCC
LRRC8A_fwd	CAGTCCTGTGCATTCAGGTGG
LRRC8A_rev	GAATCATGGTTCAACCCCAAGG
HOXB13_fwd	AAGGCAGCATTTGCAGACTCC
HOXB13_rev	GGTGATGAACTTGTTAGCCGC
PTGER3_fwd	CTTCGAAAGTTTTGCCAGGAGG
PTGER3_rev	GGAGCTTCCAGTGATGTGATCC

DKK1_fwd	TGACAACTACCAGCCGTACC
DKK1_rev	TGCAGGCGAGACAGATTTGC
TIE1_fwd	ACCACCAAGAGTGATGTCTGG
TIE1_rev	TTCATAGAGCTCGGCACAGG
UBE2B_fwd	TTTGGACCAGAAGGGACACC
UBE2B_rev	GCTACCATCAGCATACACATTTGG
APEX1_fwd	CTCCTTCGGACAAGGAAGGG
APEX1_rev	TGCTCCTCATCGCCTATGCC
RNF138_fwd	CCTGTCAGCACGTCAATAGG
RNF138_rev	TGTTACAGTGATCCAGTAAACGC
MAPK1_fwd	TAACGTTCTGCACCGTGACC
MAPK1_rev	TGGACTTGGTGTAGCCCTTGG
CYLD_fwd	TCACTTCCCAAAGACTTACCCG
CYLD_rev	GCCATTCTGACCACCATCCC
PROM1_fwd	TAGCAACCCTGAACTGAGGC
PROM1_rev	AGGGATTGATAGCCCTGTTGG
NFE2L2_fwd	CAACTACTCCCAGGTTGCCC
NFE2L2_rev	CAAGTGACTGAAACGTAGCCG
BAG1_fwd	AGATTGACACACTGATCCTGCC
BAG1_rev	TCTGCTACACCTCACTCGGC
BECN1_fwd	CGATGGTAGTTCTGGAGGC
BECN1_rev	TAGACCCTTCCATCCCTCGG
BAD_fwd	AGTTTGTGGACTCCTTTAAGAAGGG
BAD_rev	ACCAGGACTGGAAGACTCGC
VIM_fwd	CGATGTGGATGTTTCCAAGCC
VIM_rev	TCAGAGAGGTCAGCAAACTTGG
HMGB1_fwd	TGGATAGGGTGGTGTGGAGG
HMGB1_rev	GCCCATGTTTAGTTATTTTTCAGCC
RUNX3_fwd	CCGGGAATCCAAATTCTTGGTAGC
RUNX3_rev	GATGCTGTTCGATGCCATGC
SFN_fwd	CGCTGTTCTTGCTCCAAAGG
SFN_rev	ATGACCAGTGGTTAGGTGCG
PYCARD_fwd	TCTACCTGGAGACCTACGGC
PYCARD_rev	TATAAAGTGCAGGCCCTGGTGC
SIRT1_fwd	CTCTTTTCCTCCGTCCGTGG
SIRT1_rev	CCGAACAGAAGGTTATCTGGC
H19 _fwd	GAGTCTGGCAGGAGTGATGACG
H19 _rev	CCTGTAACCAAAAGTGACCGGG
GAPDH fwd	AAGGTCGGAGTCAACGGATTT
GAPDH rev	ACCAGAGTTAAAAGCAGCCCTG
U6 fwd	CTCGCTTCGGCAGCACA
U6 rev	AACGCTTCACGAATTTGCGT
